# Long-term results and recurrence patterns from SCOPE-1: a phase II/III randomised trial of definitive chemoradiotherapy **+**/**−** cetuximab in oesophageal cancer

**DOI:** 10.1038/bjc.2017.21

**Published:** 2017-02-14

**Authors:** T Crosby, C N Hurt, S Falk, S Gollins, J Staffurth, R Ray, J A Bridgewater, J I Geh, D Cunningham, J Blazeby, R Roy, T Maughan, G Griffiths, S Mukherjee

**Affiliations:** 1 Velindre Cancer Centre, Velindre Hospital, Cardiff CF14 2TL, UK; 2 Wales Cancer Trials Unit, Cardiff University, Cardiff CF14 4YS, UK; 3 Bristol Haematology and Oncology Centre, University Hospitals Bristol NHS Foundation Trust, Bristol BS2 8ED, UK; 4 North Wales Cancer Treatment Centre, Conwy and Denbighshire NHS Trust, Rhyl LL18 5UJ, UK; 5 UCL Cancer Institute, University College London, London WC1E 6BT, UK; 6 Queen Elizabeth Hospital, University Hospitals Birmingham NHS Foundation Trust Birmingham B15 2GW, UK; 7 The Royal Marsden Hospital NHS Foundation Trust, London SM2 5PT, UK; 8 Centre for Surgical Research, University of Bristol, Bristol BS8 2PS, UK; 9 Diana Princess of Wales Hospital, Northern Lincolnshire and Goole NHS Foundation Trust, Grimsby DN33 2BA, UK; 10 CRUK/MRC Oxford Institute for Radiation Oncology, University of Oxford, Oxford OX3 7DQ, UK; 11 Southampton Clinical Trials Unit, University of Southampton, Southampton SO16 6YD, UK

**Keywords:** oesophageal, chemoradiotherapy, phase II/III, randomised, trial

## Abstract

**Background:**

The SCOPE-1 study tested the role of adding cetuximab to conventional definitive chemoradiotherapy (dCRT), and demonstrated greater toxicity and worse survival outcomes. We present the long-term outcomes and patterns of recurrence.

**Methods:**

SCOPE-1 was a phase II/III trial in which patients were randomised to cisplatin 60 mg m^−2^ (day 1) and capecitabine 625 mg m^−2^ bd (days 1–21) for four cycles +/− cetuximab 400 mg m^−2^ day 1 then by 250 mg m^−2^ weekly. Radiotherapy consisted of 50 Gy/25# given concurrently with cycles 3 and 4. Recruitment was between February 2008 and February 2012, when the IDMC recommended closure on the basis of futility.

**Results:**

About 258 patients (dCRT=129; dCRT+cetuximab (dCRT+C)=129) were recruited from 36 centres. About 72.9% (*n*=188) had squamous cell histology. The median follow-up (IQR) was 46.2 (35.9–48.3) months for surviving patients. The median overall survival (OS; months; 95% CI) was 34.5 (24.7–42.3) in dCRT and 24.7 (18.6–31.3) in dCRT+C (hazard ratio (HR)=1.25, 95% CIs: 0.93–1.69, *P*=0.137). Median progression-free survival (PFS; months; 95% CI) was 24.1 (15.3–29.9) and 15.9 (10.7–20.8) months, respectively (HR=1.28, 95% CIs: 0.94–1.75; *P*=0.114). On multivariable analysis only earlier stage, full-dose RT, and higher cisplatin dose intensity were associated with improved OS.

**Conclusions:**

The mature analysis demonstrates that the dCRT regimen used in the study provided useful survival outcomes despite its use in patients who were largely unfit for surgery or who had inoperable disease. Given the competing risk of systemic and local failure, future studies should continue to focus on enhancing local control as well as optimising systemic therapy.

Definitive chemoradiotherapy (dCRT) is an important treatment option for localised oesophageal cancer, and is considered a standard of care for patients with oesophageal squamous cell carcinoma. Long-term outcomes from dCRT, from large prospective clinical trials incorporating a modern conformal radiotherapy delivery protocol, QOL, and a robust radiotherapy quality assurance programme (RTQA) have not previously been reported. This information is important to allow patients to make an informed choice regarding treatment options, especially as non-randomised data show that QOL may return to normal more quickly following dCRT and is better in the longer term compared with surgery (Rees *et al*, 2015).

Cancer Research UK SCOPE-1 trial was a randomised control phase II/III trial, which compared conventional cisplatin–capecitabine-based dCRT with or without addition of cetuximab. The addition of cetuximab to radiotherapy for head and neck cancer had previously shown a significant survival benefit (Bonner *et al*, 2006). Following advice from the Independent Data Monitoring Committee (IDMC), SCOPE-1 was stopped at the phase II stage after 258 patients were randomised from 36 centres across the UK between February 2008 and January 2012, because the trial met the criteria for futility. The initial publication in 2013, after a median follow-up of 16.8 months in surviving patients (IQR 11.2–24.5), reported a statistically significant overall survival (OS) detriment in the cetuximab arm (median 25.4 months (95% CIs: 20.5–37.9) *vs* 22.1 months (15.1–24.5); adjusted hazard ratio (HR) 1.53 (95% CI 1.03–2.27); *P*=0.035; Crosby *et al*, 2013).

In this final report, we have looked at the long-term outcomes in the SCOPE-1, with detailed analysis of secondary end points, patterns of failure, and implications for future research. ISRCTN: 47718479.

## Materials and methods

The trial design, treatments, eligibility criteria, and follow-up were previously reported in detail (Hurt *et al*, 2011; Crosby *et al*, 2013). In summary, the trial included patients with non-metastatic, histologically confirmed carcinoma of the oesophagus (adenocarcinoma, squamous cell, or undifferentiated; WHO status 0–1; stage I–III disease; American Joint Committee on Cancer, 6th edition) and who had been selected to receive dCRT and randomly assigned them to receive dCRT alone or dCRT with cetuximab (400 mg m^−^^2^ on day 1 followed by 250 mg m^−^^2^ weekly). Definitive chemoradiotherapy consisted of cisplatin 60 mg m^−^^2^ (day 1) and capecitabine 625 mg m^−^^2^ bd twice daily (days 1–21) for four cycles; cycles 3 and 4 were given concurrently with 50 Gy in 25 fractions of radiotherapy. Follow-up was at 24 weeks, then every 3 months during the first year, every 4 months during the second year, and yearly thereafter for a minimum of 5 years from randomisation, during which time RTOG/EORTC late radiation morbidity scores (Radiation Therapy Oncology Group, 2007) were collected. The primary end point was the proportion of patients with treatment failure-free survival (TFFS, defined as still alive with no evidence of residual malignancy in the endoscopic biopsy sample and no evidence of disease progression outside the radiotherapy field on CT scan) at week 24 for the phase II stage and OS (time to event) for the phase III stage of the trial, both of which were measured from randomisation. Secondary end points included local and distant progression-free survival (LPFS and DPFS), patterns of first progression, and late toxicity. Distant progression-free survival was defined as time to progression with metastases or death by any cause. Local progression-free survival was defined as either time to progression within the RT volume (infield) or death by any cause, or outside the RT volume but within the region (outfield) or death by any cause. QOL data were collected up to 24 months and have been reported elsewhere (Rees *et al*, 2015). The trial was approved by a UK multi-centre ethics committee and obtained individual informed consent from all participants. The trial protocol and radiotherapy guideline can be found in Supplementary Materials 1 and 2, respectively.

All statistical analyses were pre-planned and conducted using Stata SE 14 (StataCorp, College Station, TX, USA). All analyses were performed according to the intention-to-treat principle and included all randomly assigned patients. Follow-up time distributions were estimated using the reverse Kaplan–Meier method (Schemper and Smith, 1996) with patients censored at date of death or last trial assessment. We calculated % of total dose (actual total dose divided by protocol total dose) and % dose intensity (actual dose intensity (dose per unit time) divided by protocol dose intensity) for each protocol drug as measures of compliance. As has been done elsewhere (Loibl *et al*, 2011), patients who progressed or died during the treatment period had denominators calculated up to the point where they progressed or died. Likewise for radiotherapy we calculated % of full protocol dose received by each patient and for those who progressed or died during the treatment period, the denominator was calculated up to the point where they progressed or died. We calculated survival from date of randomisation to when an event occurred, that is, progression or any death for PFS, and any death for OS. Patients who were event free were censored at the time they were last known to be event free. We estimated event time distributions with the Kaplan–Meier method and compared OS and PFS with HRs from Cox regression in univariable models and multivariable models. In the multivariable models we included all variables thought *a priori* to potentially have a prognostic effect and included recruitment centre as a random frailty effect. The consistency of the main univariable treatment arm effect was assessed across subgroups using HR plots and the significance of treatment arm-subgroup variable interaction terms in Cox models. We tested the proportional hazards assumption of each model with Cox-Snell residuals and Schoenfeld's global test. We did not adjust for having previously looked at the HRs, as survival was a secondary end point to the phase II trial.

## Results

### Study population

**Figure 1:**
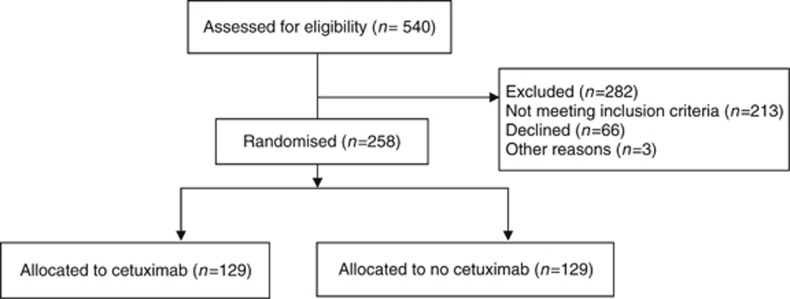
**Flow diagram of trial participants.**

The study population was described in detail in the first report and Figure 1; (Crosby *et al*, 2013). In summary, median age was 66.7 (IQR: 60.9–72.9), 56.2% (*n*=145) were male, 50.8% (*n*=131) were WHO PS 0 (rather than 1), 60.1% (*n*=155) had stage III disease, 72.9% (*n*=188) had squamous cell type, and the median disease length was 5 cm (IQR: 4–7.5). The main reason for no surgery was local extent of disease (47.3%, *n*=122). T stage: T1 3.5% (*n*=9), T2 18.2% (*n*=47), T3 63.2% (*n*=163), T4 15.1% (*n*=39). N stage: N0 33.3% (*n*=86), N1 66.7% (*n*=172).

**Figure 2:**
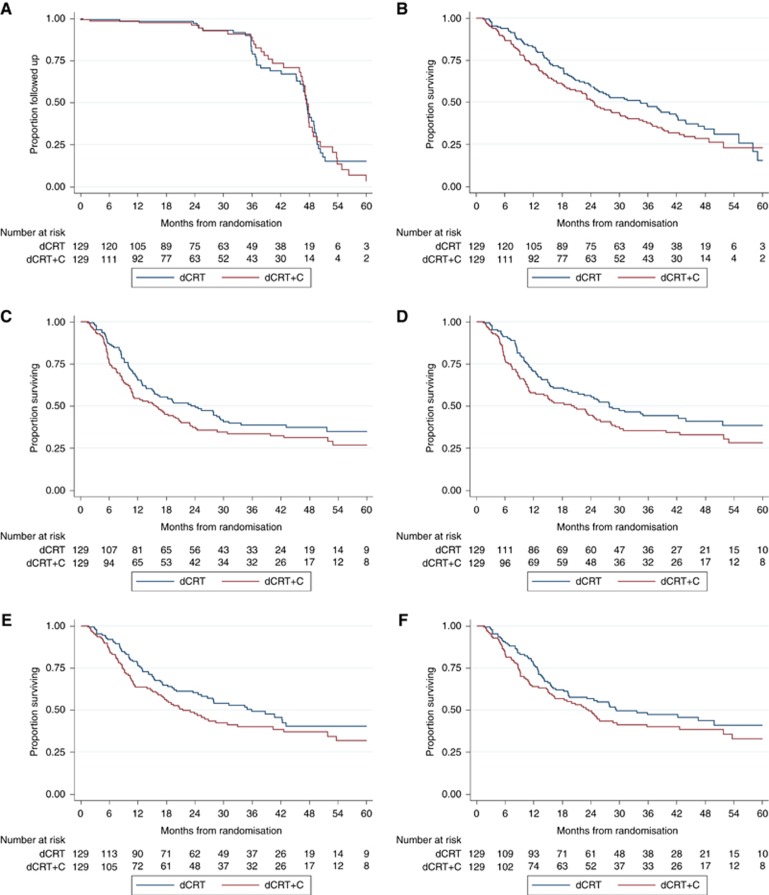
**Kaplan–Meier curves of follow-up and survival by treatment group.**(**A**) Follow-up (reverse Kaplan–Meier). (**B**) Overall survival. (**C**) Progression-free survival. (**D**) Local progression-free survival (infield). (**E**) Local progression-free survival (outfield). (**F**) Distant progression-free survival.

Median follow-up was 46.2 (IQR: 35.9–48.3) months for surviving patients. This was balanced across trial arms with median times of 45.2 (IQR: 35.8–48.2) and 46.8 (IQR: 36.4–48.8) months in the dCRT only and dCRT+C arms, respectively (Figure 2A).

### Compliance

Significantly more patients completed all four cycles of cisplatin and capecitabine in the dCRT-only group (Crosby *et al*, 2013). More patients in the dCRT group received ⩾75% of the total protocol dose of capecitabine (72.1 (93 out of 129) *vs* 62.8% (81 out of 129), *χ*^2^=2.542, *P*=0.111). The proportion of patients receiving ⩾75% of the full protocol cisplatin dose intensity was similar in both trial arms (dCRT: 71% (91 out of 129); dCRT+C: 71% (92 out of 129); Supplementary Figure S1). This was mainly due to a larger proportion of patients in the dCRT+C group receiving full cisplatin dose in the second cycle of chemotherapy. Significantly more patients in the dCRT group received the full protocol dose of radiotherapy (90.7 (117 out of 129) *vs* 79.1% (102 out of 129), *χ*^2^=6.796, *P*=0.009). Six patients died (three due to oesophageal cancer, one sepsis, one stroke, and one vascular disorder of intestine) prior to the end of the treatment period.

### Toxicities

The RTOG/EORTC late radiation morbidity scores are shown in Supplementary Table S1. Assessment completion rates were high with scores being obtained in between 91.5% (214 out of 234) at 6 months and 87.9% (123 out of 140) at 24 months. Rates of oesophageal late radiation morbidity were initially slightly higher in the dCRT arm reflecting the higher doses achieved in that arm. However, across arms the rates of worst grade of any residual toxicity were: 9.3% (20 out of 214) grade 1, 6.5% (14 out of 214) grade 2, and 0.5% (1 out of 214) grade 3 at 6 months and this dropped to only 2.4% (3 out of 123) grade 1 at 24 months.

### Overall survival

The mature OS analysis (Table 1 and Figure 2B) demonstrates the median survival in the dCRT arm to be 34.5 months (95% CI: 24.7–42.3) *vs* 24.7 months (95% CI: 18.6–31.3) in the dCRT+C arm. With prolonged follow-up, the HR was no longer statistically significant in either univariable or multivariable analysis (1.25 (95% CIs: 0.93–1.69, *P*=0.137) and 1.15 (95% CIs: 0.84–1.57, *P*=0.388), respectively). Three-year OS was 47.2% (95% CIs: 38.2–55.7) in the dCRT-only arm and 37.6% (95% CIs: 29.1–46.0) in the dCRT+C arm. In patients receiving conventional dCRT: with squamous cell subtype, the median OS was 35.9 months (95% CI: 24.7–44.0; *n*=96, 3-year OS: 47.8% (95% CI: 37.1–57.6%)); in the adenocarcinoma subtype the median OS was 25.8 months (95% CIs: 12.5–46.6; *n*=32; 3-year OS: 43.8% (95% CIs: 26.5–59.8%)).

**Figure 3:**
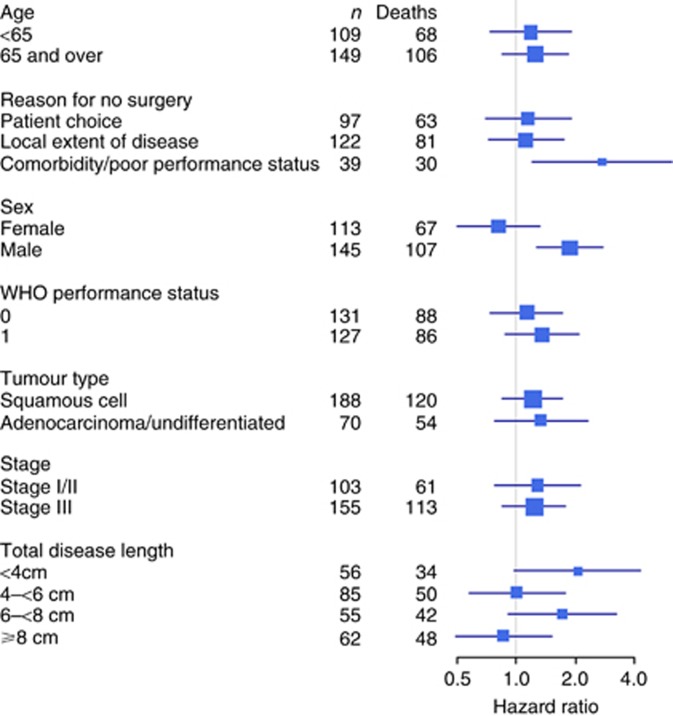
**Hazard ratio plots for treatment effect on overall survival by baseline characteristics (HR>1 favours dCRT only).**

Higher stage, less than full protocol radiotherapy dose, and lower cisplatin dose intensity were associated with worse survival in multivariable analysis (Table 1). The better survival in the dCRT arm was consistent across most subgroups of baseline characteristics other than in females and those with disease length ⩾8 cm (Figure 3) and only the sex treatment arm interaction term was significant in Cox models (*z*=2.58, *P*=0.010). In females the HR for treatment effect was 0.81 (95% CI: 0.50–1.31), whereas in males it was 1.87 (95% CIs: 1.26–2.77). A sensitivity analysis that only included patients who were alive at the end of the treatment period gave the same findings.

TTFS at 24 weeks continued to be highly prognostic of OS with the failures (*n*=68) having a median OS of 8.3 months (95% CIs: 6.7–12.5) and those failure free (*n*=172) having a median OS of 42.3 months (95% CIs: 35.9–48.8).

In the dCRT+C arm only, there was no difference in survival between those with any grade 3+dermatological toxicity during treatment (*n*=28) when compared to those without (*n*=101) (HR=1.03, 95% CI: 0.63–1.69, *P*=0.895).

### Progression-free survival

The PFS analysis (Table 2 and Figure 2C) suggests that the median PFS was still higher in the dCRT-only arm (24.1 months (95% CIs: 15.3–29.9) *vs* 15.9 months (95% CIs: 10.7–20.8)), although the HR was still not significant on either univariable or multivariable analysis (HR=1.28; 95% CIs: 0.94–1.75; *P*=0.114 and HR=1.20; 95% CIs: 0.87–1.66; *P*=0.220, respectively). Higher stage, male sex, and lower cisplatin dose intensity were associated with worse PFS in multivariable analysis. A sensitivity analysis that only included patients who were alive at the end of the treatment period gave the same findings.

Similar results were seen in LPFS (infield), LPFS (outfield), and DPFS (Figure 2D–F). The median LPFS (infield) was better in the dCRT arm and this approached statistical significance: 27.9 months (95% CIs: 19.2–51.7) *vs* 20.0 months (95% CIs: 11.1–26.1), HR=1.36 (95% CIs: 0.99–1.87, *P*=0.062). Differences in LPFS (outfield) and DPFS also favoured the dCRT arm although with no statistical significance; median LPFS (outfield): 35.9 months (24.7–62.1) *vs* 21.5 months (16.3–33.0), HR=1.32 (0.94–1.85), *P*=0.112; median DPFS: 29.3 months (19.3–60.6) *vs* 23.5 months (15.9–35.8), HR=1.24 (0.89–1.73), *P*=0.207. In the multivariable models for LPFS (infield), LPFS (outfield), and DPFS, only less than full protocol radiotherapy dose and lower cisplatin dose intensity remained significant at the *P*<0.05 level (data not shown).

### Patterns of first progression

The patterns of first progression are shown in Table 3. There were 77 progressions prior to death or date last seen in the dCRT arm and 85 in the dCRT+C group. More progressions involved a distant progression only (76 out of 162 (46.9%)) compared to loco-regional progression only (57 out of 162 (35.2%)). In the dCRT arm, out of 38 patients with a loco-regional progression, 31 progressed within the RT field compared to 40 out of 48 in the dCRT+C group (81.6% *vs* 83.3%, respectively, *χ*^2^=0.0453, *P*=0.831). Very similar patterns were seen in both squamous cell and adenocarcinoma/undifferentiated tumours (Supplementary Table S2).

### Causes of death

At the time of analysis, 84 (65.1%) patients had died (69 (53.5%) of oesophageal cancer) in the dCRT arm and 90 (69.8%) patients had died (78 (60.5%) of oesophageal cancer) in the dCRT+C arm (Supplementary Table S3).

## Discussion

These long-term data, analysed after death of nearly two-thirds of the patients in both arms and after a median follow-up of 46.2 months in surviving patients, show that median OS in the dCRT arm (34.5 months (95% CI: 24.7–42.3)) is better than initially reported. Although survival in the standard dCRT arm remained superior to the cetuximab arm, the difference was no longer statistically significant. Local progression within radiation field was higher in the cetuximab arm, which approached statistical significance (*P*=0.062); full-dose radiotherapy and a cisplatin dose intensity of >75% were associated with improved survival in the multivariable model. Long-term residual treatment toxicity was very low and TFFS remained a highly significant surrogate for OS. A difference in treatment effect was found by sex with the better survival in the dCRT arm not being seen in females; however, this was an exploratory analysis for which we do not have a hypothesis and may be due to chance, therefore further corroboration is needed (Wallach *et al*, 2016).

At the time of initiating this study, there was significant interest in combining radiotherapy with cetuximab following reported survival advantage for this combination in a randomised controlled trial in head and neck cancer, which showed near doubling of median OS (54 *vs* 28 months, *P*=0.02) and improved 3-year OS (57 *vs* 44% Bonner *et al*, 2006). Preclinical data suggested radioresistance in EGFR overexpressing head and neck cell lines and this radioresistance could be reversed through EGFR blockade (Akimoto *et al*, 1999; Huang *et al*, 1999, 2002; Bonner *et al*, 2000; Milas *et al*, 2000, 2004; Harari and Huang, 2001; Nasu *et al*, 2001; Shintani *et al*, 2003). Subsequent to SCOPE-1, several other studies have reported outcomes from EGFR inhibition and CRT in oesophageal cancer (Ruhstaller *et al*, 2011; Becerra *et al*, 2013; Meng *et al*, 2013; Ubink *et al*, 2014). Most notably, the RTOG 0436 trial randomised 344 patients to weekly paclitaxel, cisplatin with or without cetuximab and 50.4 Gy of radiotherapy. The study failed to demonstrate improvements in OS, local control, or clinical complete response rate (Suntharalingam *et al*, 2014).

The inability to deliver adequate doses of standard chemoradiotherapy treatment has been a consistent feature of randomised trials of cetuximab in head and neck, and GI cancer which may have contributed to inferior outcomes in the cetuximab arms (Maughan *et al*, 2011; Waddell *et al*, 2013; Ang *et al*, 2014). In RTOG 0522, a trial designed to test the benefits for the addition of cetuximab to chemoradiotherapy in squamous cell head and neck cancer, patients receiving cetuximab experienced more interruptions to radiotherapy treatment and no survival advantage. The findings from the SCOPE-1 trial are consistent with these observations.

Despite the lack of benefit from the addition of cetuximab, it is important to report long-term outcomes of dCRT trials, as mature survival data using this modality in oesophageal cancer are lacking. Although a significant proportion of patient was elderly (39% above the age of 70 years), the majority had stage 3 disease (60%), and most patients were unsuitable for surgery due to advanced local disease (47%) and/or co-morbidities (16%); the OS in the standard arm of this study is among the best in published literature on dCRT (Cooper *et al*, 1999; Stahl *et al*, 2005; Bedenne *et al*, 2007; Herskovic *et al*, 2012; Conroy *et al*, 2014). The encouraging long-term survival seen in SCOPE-1 reassures us that dCRT is not an unreasonable alternative to surgery, particularly in patients with borderline fitness where surgery is considered a high-risk procedure. In addition, local recurrence, often raised as an important weakness of dCRT over surgery, was lower in this trial when compared with other historic reports of dCRT (Minsky *et al*, 2002; Denham *et al*, 2003; Herskovic *et al*, 2012).

So what factors are likely to have contributed to the good outcome seen in the dCRT arm of this trial compared to historic studies? For the first time in the United Kingdom, this trial introduced a detailed protocol for staging patients with oesophageal cancer due to undergoing non-surgical treatment, thereby improving patient selection – all eligible patients had an EUS and 85% were staged with 18F-FDG CT-PET. It also introduced what, at the time, was advanced, conformal radiotherapy treatment by way of a detailed radiotherapy planning guidance document together with a RTQA programme (Gwynne *et al*, 2013). The authors believe such RTQA programmes, which included pre-trial test case and real-time radiotherapy planning reviews in a proportion of patients, would have contributed to the improved outcomes and should become a mandatory component of future radiotherapy trials (Ibbott *et al*, 2013).

Treatments after the end of trial chemoradiotherapy were not accounted for in the analysis and therefore the impact of second- and subsequent-line therapies on OS cannot be ascertained. Moreover, no imaging was mandated after 24 weeks and detection of progression relied on clinical assessment at follow-up visits – mandating serial imaging may have led to earlier detection of disease progression and therefore earlier institution of second-line treatment. Nevertheless, this mirrors standard of care for this patient population and there is little evidence for a survival advantage for any treatment intervention over another post dCRT.

The failure of SCOPE-1 and other cetuximab-based studies argues against the non-selective use of biological agents in oncology trials, highlighting the potential for harm and not just lack of efficacy. The use of a less toxic chemotherapy backbone may also allow incorporation of novel agents into dCRT regimens without compromising dose intensity of conventional treatment. The weekly carboplatin-/paclitaxel-based neo-adjuvant CRT regimen, as established by the CROSS trial Shintani *et al*, 2003, reported low incidence of treatment-related toxicity and an impressive pathological complete response of about 50% in the squamous cell subtype – such low-toxicity regimens should be explored in the context of dCRT trials in oesophageal cancer.

In summary, we concur with our previous report that cetuximab should not be used as standard with CRT in the treatment of oesophageal cancer. This mature analysis highlighted the excellent survival and low rate of long-term toxicity of standard dCRT, which taken in conjunction with the early return of quality of life (Rees *et al*, 2015), suggests dCRT is an effective treatment modality. Given the competing risks of systemic and local failure, efforts should continue to maximise local control through radiotherapy dose intensification (in the context of modern day radiotherapy planning and delivery) and to identify more effective systemic treatments for those patients who do not, or are unlikely to, respond to conventional dCRT. Such an approach, including radiotherapy dose escalation and individualisation of systemic therapy through early PET response will be pursued in Cancer Research UK-funded SCOPE-2 trial, currently in set up in the United Kingdom (EudraCT No: 2015–001740–11).

## Figures and Tables

**Table 1:** Univariable and multivariable Cox regression analysis of OS

	**OS (months)**			**Univariable**			**Multivariable**		
	***n***	**Median**	**95% CIs**	**HR**	**95% CIs**	***P*****-value**	**HR**	**95% CIs**	***P*****-value**
**Trial arm**									
CRT only	129	34.5	24.7–42.3	1			1		
CRT+cetuximab	129	24.7	18.6–31.3	1.25	0.93–1.69	0.137	1.15	0.84–1.57	0.388
**Age**									
<65	109	36.7	24.9–43.6	1			1		
⩾65	149	24.5	19.7–30.1	1.36	1.00–1.85	0.047	1.32	0.95–1.84	0.093
**Reason for no surgery**									
Patient choice	97	31.3	24.0–44.0	1			1		
Local extent of disease	122	24.7	18.6–34.5	1.2	0.86–1.68	0.276	0.97	0.67–1.41	0.875
Comorbidity/poor PS	39	31.6	14.8–42.7	1.25	0.81–1.94	0.318	0.98	0.58–1.67	0.949
**Sex**									
Female	113	34.6	24.7–48.8	1			1		
Male	145	24.9	19.6–31.6	1.44	1.06–1.95	0.02	1.35	0.96–1.90	0.083
**WHO status**									
0	131	30.3	24.0–38.4	1			1		
1	127	24.9	19.2–34.3	1.14	0.84–1.53	0.405	1.07	0.77–1.50	0.675
**Stage**									
I or II	103	42.4	31.3–49.9	1			1		
III	155	24	18.6–26.8	1.65	1.20–2.27	0.002	1.52	1.05–2.21	0.028
**Tumor type**									
Squamous	188	28.4	24.0–38.0	1			1		
Adeno/undiff	70	24.9	15.9–35.1	1.24	0.90–1.72	0.192	1.01	0.67–1.52	0.961
**Full radiation dose**									
Yes	217	34.3	25.8–39.1	1			1		
No	41	10	5.9–18.4	3.19	2.17–4.70	0	2.06	1.21–3.49	0.008
**Cisplatin intensity**									
⩾75%	182	35.9	27.2–42.4	1			1		
<75%	76	16.2	12.5–20.8	2.18	1.59–2.99	0	1.8	1.12–2.89	0.016
**Cape/5FU intensity**									
⩾75%	172	34.5	25.4–39.4	1			1		
<75%	86	20	15.4–24.7	1.66	1.22–2.26	0.001	0.85	0.54–1.34	0.493
**Total disease length**									
<4 cm	56	36	24.7–58.0	1			1		
⩾4–<6 cm	85	37.9	24.0–49.9	0.98	0.63–1.52	0.928	0.98	0.62–1.55	0.928
⩾6–<8 cm	55	24.9	18.6–40.3	1.46	0.92–2.33	0.107	1.2	0.72–2.00	0.489
⩾8 cm	62	18.4	14.9–27.8	1.84	1.17–2.89	0.009	1.5	0.91–2.50	0.115

Abbreviations: 5FU=Fluorouracil; CI=confidence interval; CRT=chemoradiotherapy; HR=hazard ratio; OS=overall survival; PS=performance status; WHO=World Health Organisation.

**Table 2:** Univariable and multivariable Cox regression analysis of PFS

	**PFS (months)**			**Univariable**			**Multivariable**		
	***n***	**Median**	**CI**	**HR**	**95% CIs**	***P*****-value**	**HR**	**95% CIs**	***P*****-value**
**Trial arm**									
CRT only	129	24.1	15.3–29.9	1			1		
CRT+cetuximab	129	15.9	10.7–20.8	1.29	0.94–1.75	0.111	1.22	0.89–1.69	0.22
**Age**									
<65	109	24.2	15.4–43.2	1			1		
⩾65	149	15.5	11.3–20.6	1.33	0.97–1.83	0.076	1.3	0.92–1.83	0.134
**Reason for no surgery**									
Patient choice	97	20.8	15.9–28.6	1			1		
Local extent of disease	122	15.3	11.5–24.2	1.15	0.82–1.62	0.409	0.93	0.64–1.35	0.7
Comorbidity/poor PS	39	17.6	9.3–51.7	1.15	0.71–1.85	0.566	0.78	0.44–1.36	0.378
**Sex**									
Female	113	22.6	14.9–51.8	1			1		
Male	145	15.9	11.3–22.9	1.44	1.05–1.97	0.024	1.43	1.02–2.01	0.039
**WHO status**									
0	131	22.6	15.3–28.6	1			1		
1	127	15.5	10.8–21.1	1.14	0.84–1.55	0.412	1.23	0.88–1.72	0.233
**Stage**									
I or II	103	28.6	16.7–60.6	1			1		
III	155	14.2	10.9–20.8	1.5	1.09–2.08	0.014	1.48	1.02–2.15	0.038
**Tumor type**									
Squamous	188	20	15.1–27.8	1			1		
Adeno/undiff	70	15.3	10.4–20.6	1.32	0.94–1.85	0.115	1.1	0.72–1.69	0.652
**Full radiation dose**									
Yes	219	20	15.9–27.9	1			1		
No	39	6.1	3.4–11.0	2.59	1.64–4.08	0	1.71	0.97–3.00	0.062
**Cisplatin intensity**									
⩾75%	183	22.9	16.4–29.9	1			1		
<75%	75	10.7	8.4–15.6	1.99	1.42–2.77	0	1.97	1.24–3.12	0.004
**Cape/5FU intensity**									
⩾75%	174	20.8	15.5–28.6	1			1		
<75%	84	13.8	8.2–20.6	1.44	1.04–2.00	0.029	0.82	0.53–1.28	0.38
**Total disease length**									
<4 cm	56	23.5	11.1-.	1			1		
⩾4–<6 cm	85	23.2	15.4–62.3	0.96	0.61–1.53	0.877	0.89	0.55–1.43	0.626
⩾6–<8 cm	55	13.8	8.4–25.4	1.51	0.93–2.45	0.095	1.37	0.80–2.34	0.252
⩾8 cm	62	12.8	10.0–20.0	1.57	0.99–2.51	0.058	1.25	0.74–2.09	0.405

Abbreviations: 5FU=Fluorouracil; CI=confidence interval; CRT=chemoradiotherapy; HR=hazard ratio; PFS=progression-free survival; PS=performance status; WHO=World Health Organisation.

**Table 3:** Patterns of first progression in relation to radiation target volumes (number of events) by trial arm

	**dCRT**						**dCRT+C**					
	**Infield**		**Outfield**		**Both**		**Infield**		**Outfield**		**Both**	
	***n***	**%**	***n***	**%**	***n***	**%**	***n***	**%**	***n***	**%**	*n*	%
Loco-regional only	19	24.7	3	3.9	5	6.5	19	22.4	5	5.9	6	7.1
Loco-regional plus distant	5	6.5	4	5.2	2	2.6	10	11.8	3	3.5	5	5.9
Distant only			39	50.6					37	43.5		
Total	24	31.2	46	59.7	7	9.1	29	34.1	45	52.9	11	12.9

Abbreviation: dCRT=definitive chemoradiotherapy. Note: Percentages calculated using total number of progressions (77 in dCRT, 85 in dCRT+C) as denominator.
