# Supplementary material for: Long-term results and recurrence patterns from SCOPE-1: a phase II/III randomised trial of definitive chemoradiotherapy +/− cetuximab in oesophageal cancer
Source: Br J Cancer. 2017 Feb 14;116(6):709–16. doi: 10.1038/bjc.2017.21 (PMC5355926; doi:10.1038/bjc.2017.21)
Supplement: Supplementary Information [file bjc201721x1.docx]

Supplementary Figure S1. Chemotherapy total dose and dose intensity

| 1. Cisplatin total dose   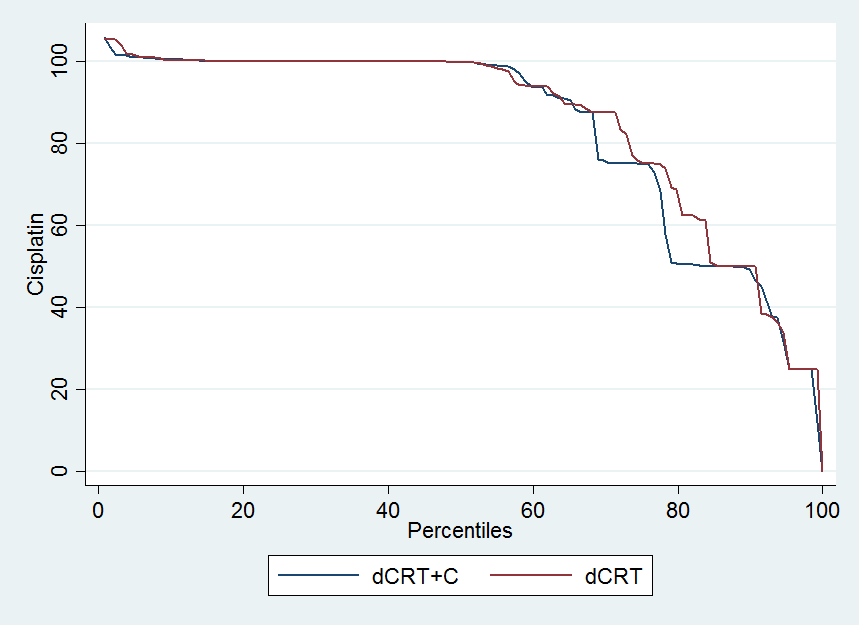 | 1. Cisplatin dose intensity   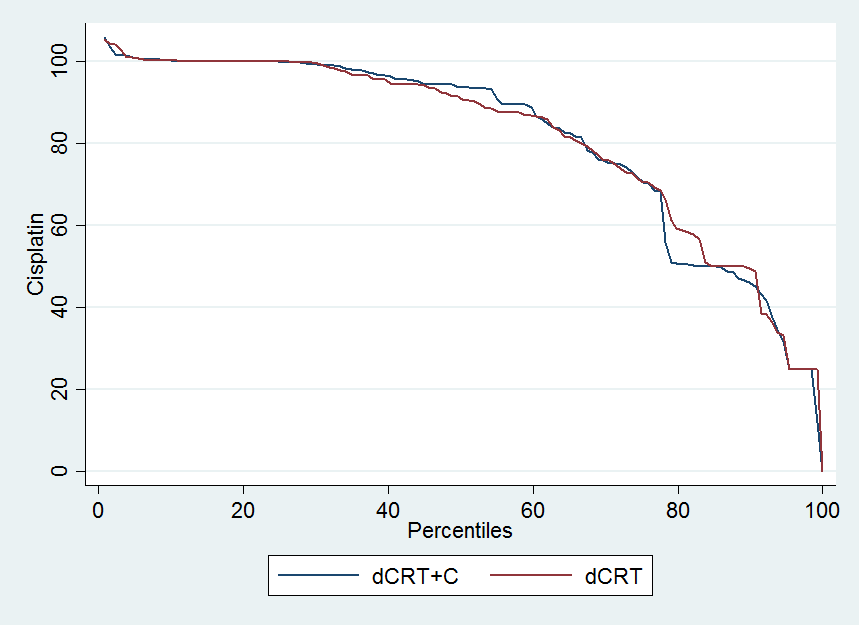 |
| --- | --- |
| 1. Cape/5FU total dose   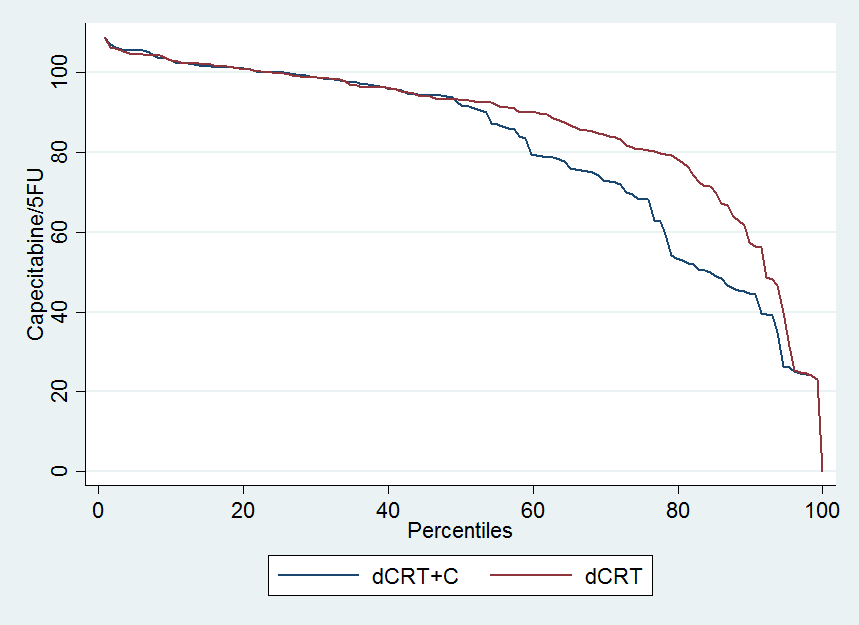 | 1. Cape/5FU dose intensity   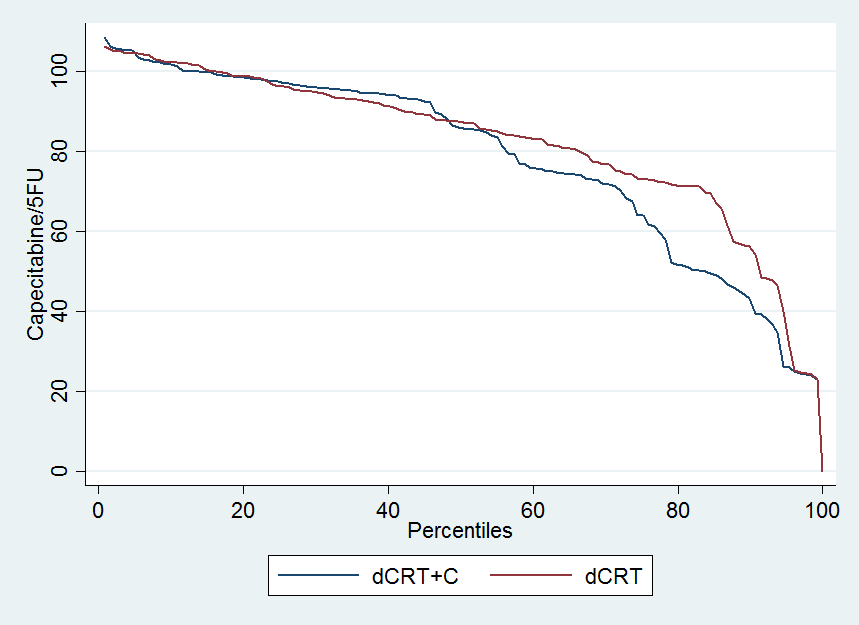 |
| 1. Cetuximab total dose   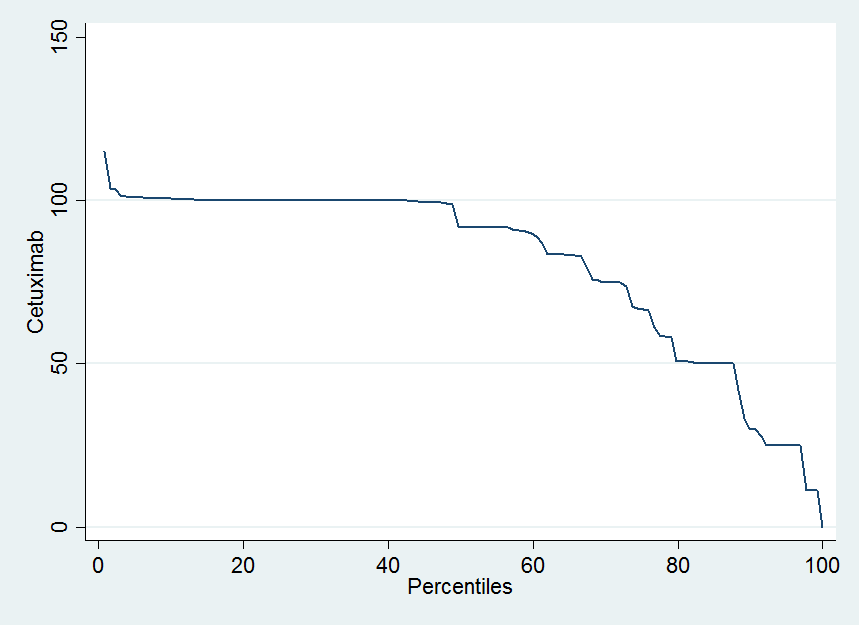 | 1. Cetuximab dose intensity   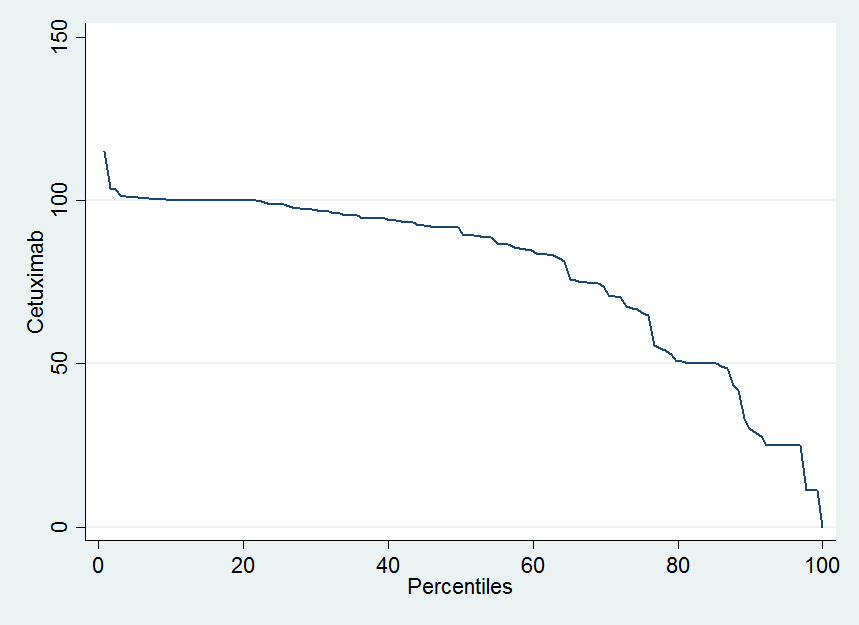 |
