# Supplementary material for: Long-term results and recurrence patterns from SCOPE-1: a phase II/III randomised trial of definitive chemoradiotherapy +/− cetuximab in oesophageal cancer
Source: Br J Cancer. 2017 Feb 14;116(6):709–16. doi: 10.1038/bjc.2017.21 (PMC5355926; doi:10.1038/bjc.2017.21)
Supplement: Supplementary Information [file bjc201721x2.docx]

Supplementary Table S1. The RTOG/EORTC late radiation morbidity scores up to 2 years after randomization in all patients

|  |  | **dCRT+C (n=129)** | | | | | | | | | | **dCRT (n=129)** | | | | | | | | | |
| --- | --- | --- | --- | --- | --- | --- | --- | --- | --- | --- | --- | --- | --- | --- | --- | --- | --- | --- | --- | --- | --- |
|  |  | **6 months** | | **9 months** | | **12 months** | | **16 months** | | **24 months** | | **6 months** | | **9 months** | | **12 months** | | **16 months** | | **24 months** | |
|  |  | **n** | **%** | **n** | **%** | **n** | **%** | **n** | **%** | **n** | **%** | **n** | **%** | **n** | **%** | **n** | **%** | **n** | **%** | **n** | **%** |
| Patients died before timepoint |  | 16 | 12.4 | 26 | 20.2 | 36 | 27.9 | 48 | 37.2 | 64 | 49.6 | 8 | 6.2 | 18 | 14.0 | 24 | 18.6 | 36 | 27.9 | 54 | 41.9 |
| Assessable patients |  | 113 | 87.6 | 103 | 79.8 | 93 | 72.1 | 81 | 62.8 | 65 | 50.4 | 121 | 93.8 | 111 | 86.0 | 105 | 81.4 | 93 | 72.1 | 75 | 58.1 |
| Patient withdrew before timepoint |  | 7 | 5.4 | 9 | 7.0 | 4 | 3.1 | 7 | 5.4 | 4 | 3.1 | 4 | 3.1 | 7 | 5.4 | 6 | 4.7 | 5 | 3.9 | 2 | 1.6 |
| Patient did not attend or toxicity assesssment not done |  | 4 | 3.1 | 7 | 5.4 | 5 | 3.9 | 6 | 4.7 | 5 | 3.9 | 5 | 3.9 | 7 | 5.4 | 4 | 3.1 | 7 | 5.4 | 6 | 4.7 |
| Assessment done |  | 102 | 90.3 | 87 | 84.5 | 84 | 90.3 | 68 | 84.0 | 56 | 86.2 | 112 | 92.6 | 97 | 87.4 | 95 | 90.5 | 81 | 87.1 | 67 | 89.3 |
| Tissue | Grade |  |  |  |  |  |  |  |  |  |  |  |  |  |  |  |  |  |  |  |  |
| Skin | 1 | 4 | 3.9 | 3 | 3.4 | 2 | 2.4 | 1 | 1.5 | 0 | 0.0 | 4 | 3.6 | 2 | 2.1 | 0 | 0.0 | 2 | 2.5 | 1 | 1.5 |
|  | 2+ | 0 | 0.0 | 0 | 0.0 | 0 | 0.0 | 0 | 0.0 | 0 | 0.0 | 0 | 0.0 | 0 | 0.0 | 0 | 0.0 | 0 | 0.0 | 0 | 0.0 |
| Oesophagus | 1 | 3 | 2.9 | 2 | 2.3 | 2 | 2.4 | 2 | 2.9 | 1 | 1.8 | 9 | 8.0 | 5 | 5.2 | 1 | 1.1 | 2 | 2.5 | 1 | 1.5 |
|  | 2+ | 5 | 4.9 | 1 | 1.1 | 2 | 2.4 | 0 | 0.0 | 0 | 0.0 | 5* | 4.5 | 4* | 4.1 | 4** | 4.2 | 1 | 1.2 | 0 | 0.0 |
| Worst of heart, lung, mucous membrane, spinal cord | 1 | 3 | 2.9 | 1 | 1.1 | 0 | 0.0 | 2 | 2.9 | 1 | 1.8 | 6 | 5.4 | 1 | 1.0 | 0 | 0.0 | 1 | 1.2 | 1 | 1.5 |
|  | 2+ | 3 | 2.9 | 0 | 0.0 | 0 | 0.0 | 0 | 0.0 | 0 | 0.0 | 2 | 1.8 | 2* | 2.1 | 0 | 0.0 | 0 | 0.0 | 0 | 0.0 |
| Worst of any tissue | 1 | 5 | 4.9 | 4 | 4.6 | 5 | 6.0 | 4 | 5.9 | 1 | 1.8 | 15 | 13.4 | 8 | 8.2 | 3 | 3.2 | 4 | 4.9 | 2 | 3.0 |
|  | 2 | 8 | 7.8 | 1 | 1.1 | 2 | 2.4 | 0 | 0.0 | 0 | 0.0 | 6 | 5.4 | 4 | 4.1 | 2 | 2.1 | 1 | 1.2 | 0 | 0.0 |
|  | 3 | 0 | 0.0 | 0 | 0.0 | 0 | 0.0 | 0 | 0.0 | 0 | 0.0 | 1 | 0.9 | 2 | 2.1 | 2 | 2.1 | 0 | 0.0 | 0 | 0.0 |

Note: All grade 2+ are grade 2 except:

* includes 1 grade 3

** includes 2 grade 3
