# Supplementary material for: Long-term results and recurrence patterns from SCOPE-1: a phase II/III randomised trial of definitive chemoradiotherapy +/− cetuximab in oesophageal cancer
Source: Br J Cancer. 2017 Feb 14;116(6):709–16. doi: 10.1038/bjc.2017.21 (PMC5355926; doi:10.1038/bjc.2017.21)
Supplement: Supplementary Information [file bjc201721x3.docx]

Supplementary Table S2. Patterns of first progression in relation to radiation target volumes (number of events) by tumour type

|  | **Squamous cell** | | | | | | **Adenocarcinoma/Undifferentiated** | | | | | |
| --- | --- | --- | --- | --- | --- | --- | --- | --- | --- | --- | --- | --- |
|  | **Infield** | | **Outfield** | | **Both** | | **Infield** | | **Outfield** | | **Both** | |
|  | n | % | n | % | n | % | n | % | n | % | n | % |
| Loco-regional only | 29 | 25.0 | 6 | 5.2 | 6 | 5.2 | 9 | 19.6 | 2 | 4.3 | 5 | 10.9 |
| Loco-regional plus distant | 11 | 9.5 | 4 | 3.4 | 5 | 4.3 | 4 | 8.7 | 3 | 6.5 | 2 | 4.3 |
| Distant only |  |  | 55 | 47.4 |  |  |  |  | 21 | 45.7 |  |  |
| Total | 40 | 34.5 | 65 | 56.0 | 11 | 9.5 | 13 | 28.3 | 26 | 56.5 | 7 | 15.2 |

Note: Percentages calculated using total number of progressions (116 in squamous, 46 in adeno/undiff) as denominator
