# Supplementary material for: Long-term results and recurrence patterns from SCOPE-1: a phase II/III randomised trial of definitive chemoradiotherapy +/− cetuximab in oesophageal cancer
Source: Br J Cancer. 2017 Feb 14;116(6):709–16. doi: 10.1038/bjc.2017.21 (PMC5355926; doi:10.1038/bjc.2017.21)
Supplement: Supplementary Information [file bjc201721x4.docx]

Supplementary Table S3. Causes of death

| **Cause of death** | **dCRT** | | **dCRT+C** | | **Total** | |
| --- | --- | --- | --- | --- | --- | --- |
|  | **n** | **%** | **n** | **%** | **n** | **%** |
| Esophageal cancer | 69 | 53.5 | 78 | 60.5 | **147** | **57.0** |
| Acute cholecystitis | 1 | 0.8 | 0 | 0.0 | **1** | **0.4** |
| Aspiration of fluid | 1 | 0.8 | 0 | 0.0 | **1** | **0.4** |
| Atrial fibrillation/ flutter | 1 | 0.8 | 0 | 0.0 | **1** | **0.4** |
| Cerebral infarction | 1 | 0.8 | 0 | 0.0 | **1** | **0.4** |
| Congestive heart failure | 0 | 0.0 | 1 | 0.8 | **1** | **0.4** |
| Foodborne intoxication by Clostridium difficile | 0 | 0.0 | 1 | 0.8 | **1** | **0.4** |
| Influenza with pneumonia, virus not identified | 0 | 0.0 | 1 | 0.8 | **1** | **0.4** |
| Intracerebral haemorrhage | 1 | 0.8 | 0 | 0.0 | **1** | **0.4** |
| Ischaemic/pulmonary heart disease | 3 | 2.3 | 1 | 0.8 | **4** | **1.6** |
| Malignant neoplasm of bronchus or lung | 1 | 0.8 | 1 | 0.8 | **2** | **0.8** |
| Malignant neoplasm of unspecified parts of tongue | 1 | 0.8 | 0 | 0.0 | **1** | **0.4** |
| Other and unspecified cirrhosis of liver | 0 | 0.0 | 1 | 0.8 | **1** | **0.4** |
| Other interstitial pulmonary diseases with fibrosis | 0 | 0.0 | 1 | 0.8 | **1** | **0.4** |
| Pneumonia | 3 | 2.3 | 0 | 0.0 | **3** | **1.2** |
| Pulmonary embolism | 0 | 0.0 | 1 | 0.8 | **1** | **0.4** |
| Sepsis | 1 | 0.8 | 1 | 0.8 | **2** | **0.8** |
| Stroke | 0 | 0.0 | 1 | 0.8 | **1** | **0.4** |
| Unspecified intestinal obstruction | 1 | 0.8 | 0 | 0.0 | **1** | **0.4** |
| Urinary tract infection, site not specified | 0 | 0.0 | 1 | 0.8 | **1** | **0.4** |
| Vascular disorder of intestine, unspecified | 0 | 0.0 | 1 | 0.8 | **1** | **0.4** |
| **Total** | **84** | **65.1** | **90** | **69.8** | **174** | **67.4** |
